# Licochalcone A Promotes the Ubiquitination of c-Met to Abrogate Gefitinib Resistance

**DOI:** 10.1155/2022/5687832

**Published:** 2022-03-10

**Authors:** Shuangze Han, Xiaoying Li, Yu Gan, Wei Li

**Affiliations:** ^1^Department of Radiology, The Third Xiangya Hospital of Central South University, Changsha, Hunan 410013, China; ^2^Cell Transplantation and Gene Therapy Institute, The Third Xiangya Hospital, Central South University, Changsha, Hunan 410013, China

## Abstract

Met proto-oncogene (MET) amplification and tyrosine-protein kinase Met (c-Met) overexpression confer gefitinib resistance in non-small cell lung cancer (NSCLC). The natural product Licochalcone A (Lico A) exhibits a broad range of inhibitory effects against various tumors. However, the effects of Lico A on c-Met signaling and gefitinib resistance in NSCLC remain unclear. In the present study, Lico A efficiently overcame gefitinib-acquired resistance in NSCLC cells by suppressing c-Met signaling. Lico A decreased cell viability and colony formation dose-dependently and impaired in vivo tumorigenesis of gefitinib-resistant HCC827 and PC-9 cells. Furthermore, Lico A induced intrinsic apoptosis and upregulated the protein expression levels of cleaved poly (ADP-ribose) polymerase and cleaved caspase 3. Lico A promoted the interaction between c-Met and E3 ligase c-Casitas B-lineage lymphoma (Cbl), which enhanced c-Cbl-mediated c-Met ubiquitination and degradation. Depletion of c-Cbl compromised Lico A-induced c-Met ubiquitination and its inhibitory efficacy in gefitinib-resistant NSCLC cells. Taken together, the results suggest that Lico A is a promising antitumor agent that might be used to overcome c-Met overexpression-mediated gefitinib resistance in NSCLC cells.

## 1. Introduction

Non-small cell lung cancer (NSCLC) is a profoundly devastating disease that is the leading cause of cancer-associated deaths worldwide [1]. NSCLC accounts for ~85% of lung cancer cases [2]. Targetable genomic alterations in NSCLC have been examined as attractive therapeutic targets, including those occurring at epidermal growth factor receptor (EGFR), Kirsten rat sarcoma virus (KRAS), anaplastic lymphoma kinase (ALK), and phosphatidylinositol-4,5-bisphosphate 3-kinase catalytic subunit alpha (PIK3CA), and those that lead to altered production of reactive oxygen species [3–5]. Specifically, the EGFR-targeted therapies have become optimal treatment options among patients with EGFR-mutant NSCLC [6, 7]. EGFR tyrosine kinase inhibitors (TKIs) have prominently extended the overall survival and progression-free survival rates compared with conventional chemoradiotherapy for patients with advanced EGFR-mutant NSCLC [4, 7–9]. However, the acquired resistance to EGFR TKIs, gefitinib, and erlotinib remains a significant challenge [10, 11]. Resistance to EGFR TKIs can be acquired due to secondary mutations. The most common secondary mutation is the EGFR T790M mutation.The aberrant activation of tyrosine-protein kinase Met (c-Met) signaling is an EGFR-independent mechanism that confers EGFR TKIs resistance to the NSCLC cells. It enables the cell to activate downstream ERK1/2 and AKT signaling pathways [12–14]. Therefore, identifying novel antitumor agents targeting c-Met signaling may provide alternative therapeutic approaches to overcome resistance to EGFR-TKIs [15].

Licochalcone A (Lico A) is a flavonoid extracted from licorice root, demonstrating a wide range of pharmacological effects, including the anti-inflammatory and antitumor activities [16, 17]. The antitumor effects of Lico A have been documented in various types of tumors, including gastric, [18] prostate, [19] ovarian, [20] liver [21], and lung cancers [16, 22]. The Lico A promotes cell cycle arrest, induces apoptosis, and suppresses angiogenesis and metastasis [23–25]. However, the effects of Lico A on c-Met signaling and gefitinib resistance have not been fully elucidated.

In the present study, the ability of Lico A to attenuate c-Met signaling and suppress c-Met expression in a ubiquitination-dependent manner was examined. Moreover, the potential of this compound to overcome gefitinib resistance was assessed in the NSCLC cells.

## 2. Materials and Methods

### 2.1. Cell Culture and Antibodies

Human NSCLC cell HCC827 and the immortalized lung epithelial cells HBE and NL20 were obtained from American Type Culture Collection (ATCC, Manassas, VA). PC-9 cell line was a product of Sigma-Aldrich (St. Louis, MO). Both PC9 and HCC827 cells carry a Glu746-Ala750 deletion mutation in exon 19 of the EGFR [26, 27]. The gefitinib resistant cell lines, HCC827-GR, and PC-9-GR were newly established in our laboratory by exposing HCC827 or PC-9 cells to gradually increasing concentrations of gefitinib (starting at 10 nM and ending with 400 nM) for approximately 5 months. All cells were maintained at the incubator according to the standard protocols and subjected to routinely checking for mycoplasma contamination. Antibodies against p-c-Met (#3077), c-Met (#8198), cleaved-PARP (#5625), p-Akt (#4060), Bax (#14796), Akt (#4691), VDAC1 (#4661), p-ERK1/2 (#4370), *β*-actin (#3700), ERK1/2 (#9102), *α*-tubulin (#2144), cleaved-caspase 3 (#9664), cytochrome c (#4280), and ubiquitin (#3936) were obtained from Cell Signaling Technology, Inc. (Beverly, MA). The natural compound Licochalcone A (>97%) was from Selleck Chemicals (Houston, TX). Lipofectamine 2000 transfection reagent for transient transfection was purchased from Thermo Fisher Scientific (Waltham, MA).

### 2.2. MTS Assay

The cultured cells were seeded into 96-well plates (3 × 10^3^ cells/well) and treated with various concentrations of Lico A. Following 24 h of incubation, cell viability was analyzed with MTS using the CellTiter 96^®^ Aqueous One Solution kit (Promega Corporation) as determined by the manufacturer's protocol.

### 2.3. Soft Agar Assay

The soft agar assay was performed as described previously [28]. Briefly, the NSCLC cells were pretreated with Lico A and counted at a 8 × 10^3^ cells/ml density. The cells were suspended in 1 ml Eagle's basal medium containing 10% FBS and 0.3% agar and transferred into 6-well plates with a 0.6% agar base. The colony was counted following 2 weeks of culture with a light microscope.

### 2.4. Western Blot Analysis

The cells were treated with Lico A or gefitinib, and the whole-cell extract (WCE) was prepared with RIPA buffer and concentrated using the BCA protein assay (Thermo Fisher Scientific, Inc.). The Western blot analysis was performed as described previously [28]. A total of 20 *μ*g WCE was analyzed via SDS-PAGE. Subsequently, the proteins were transferred to a PVDF membrane and incubated with the primary antibody and secondary antibody sequentially. Protein expression levels were visualized using the ECL reagent (Thermo Fisher Scientific, Inc.).

### 2.5. Subcellular Fraction Isolation

The Mitochondria Isolation kit (Thermo Fisher Scientific, Inc.) was used for cytosolic and mitochondrial fraction extraction following the manufacturers' instructions.

### 2.6. Cell Transfection

Generation of stable c-Met knock out cell lines. CRISPR-Cas9-mediated gene knockout was performed as described previously (PMID: 32945473). In brief, single-guide (sg) RNAs (#1, CACATGGCAGATCGATCCAT; #2, GACCTCACCATAGCTAATCT) targeting c-Met were used for the construction of stable cell lines. In brief, the NSCLC cells were transfected with c-Met sgRNA and selected by 1 *μ*g/ml puromycin for three weeks. Single colonies were chosen for further study. For transient transfection, the siRNAs, including si-c-Cbl (sc-29242) and siCtrl (sc-37001), were purchased from Santa Cruz Biotechnology (Dallas, TX). The c-Met cDNA (RC217003) was purchased from Origene (Rockville, MD). The transient transfection was performed using the Lipofectamine 2000 (11668019, Thermo Fisher Scientific) following the manufacturer's protocol. The whole-cell extract was prepared at 2 days later after transfection.

### 2.7. RT-qPCR

The NSCLC cells were treated with Xanth for 24 h, and total RNA was extracted using the Absolutely RNA Purification Kits (Agilent). SYBR-Green Quantitative RT-qPCR Kit was used in RT-qPCR. Amplification cycles were performed as follows: Stage 1: activation, 50°C for 2 min. Stage 2: presoak, 95°C for 10 min. Stage 3: denaturation, 95°C for 15 sec; annealing: 60°C for 1 min. Stage 4: melting curve, 95°C for 15 sec, 60°C for 15 sec, and 95°C for 15 sec. The RT-qPCR results were normalized to *β*-actin. c-Met primer sequences used were forward, TGCACAGTTGGTCCTGCCATGA; reverse, CAGCCATAGGACCGTATTTCGG.

### 2.8. Xenograft Mouse Model

All in vivo animal experiments were performed in 2021, which the Institutional Animal Ethics Committee approved at The Third Xiangya Hospital, Central South University. Mice were kept in the colony cages with free access to food and tap water and standardized housing conditions (natural 12 h light-dark cycle, temperature of 23 ± 1°C, relative humidity of 55 ± 5%). The proper care and use of experimental animals, including efforts to minimize suffering and distress using analgesics or anesthetics, was based on the Guide for the Care and Use of Laboratory Animals (National Academies Press, Washington, DC). For tumor transplantation, HCC827-GR (2 × 10^6^ cells in 100 *μ*l RPMI-1640) cells were injected into the right flank of 6-week-old female athymic nude mice. Tumor volume and mouse body weight were recorded every 2 days. The tumor-bearing mice had initiated Lico A treatment when the tumor reached a maximum of 100 mm^3^. The mice were divided into two groups randomly. Lico A treatment (20 mg/kg, *n* = 5) was initiated every 2 days via intraperitoneal injection. The control group (*n* = 5) was treated with vehicle control. Animal health and behavior were monitored every 2 days. The following formula calculated tumor volume: A × B^2^ × 0.5, where A was the longest diameter of the tumor and B was the shortest diameter. B^2^ was B squared. The tumor-bearing mice were euthanized by CO_2_ when the tumor volume reached 700 mm^3^ (24 days). The fill rate of CO_2_ is 30% of the chamber volume per minute (3 liter/min), and the duration time is 5 min. Death was further confirmed by cervical dislocation.

### 2.9. Immunoprecipitation and Ubiquitination Analysis

The ubiquitination assay was performed as described previously [29]. Briefly, WCE was prepared with modified RIPA buffer (1% SDS) supplemented with protease inhibitors and N-ethylmaleimide. The lysate was boiled at 95°C for 15 min and diluted with 0.1% SDS containing RIPA buffer. Following centrifugation, the supernatant was incubated with c-Met antibody-containing protein A/G-Sepharose beads overnight at 37°C. The beads were boiled with loading buffer and subjected to immunoblotting (IB) analysis.

### 2.10. Immunohistochemical (IHC) Staining

The IHC staining was performed as described previously [29]. Briefly, the slides were dewaxed with xylene and rehydrated using gradient ethanol into double distilled water. Antigen retrieval was performed by immersing the slides into boiling sodium citrate buffer (10 mM, pH 6.0) for 10 min, followed by treatment with 3% H_2_O_2_ in methanol for an additional 10 min. The tissue slides were blocked with 50% goat serum albumin in PBS for 1 h at room temperature and incubated overnight with primary antibodies at 4°C. Following hybridization with secondary antibodies, the target proteins were visualized with the DAB Substrate kit (cat. no. 34002; Thermo Fisher Scientific, Inc.).

### 2.11. Statistical Analysis

SPSS software (version 13.0; SPSS, Inc.) was used for statistical analysis. The quantitative data are presented as the mean ± SD, and the difference was analyzed using the two-tailed Student's *t*-test or one-way ANOVA. A probability value of *P* < 0.05 was considered to indicate a statistically significant difference.

## 3. Results

### 3.1. c-Met is Required for Gefitinib Resistance

**Figure 1:**
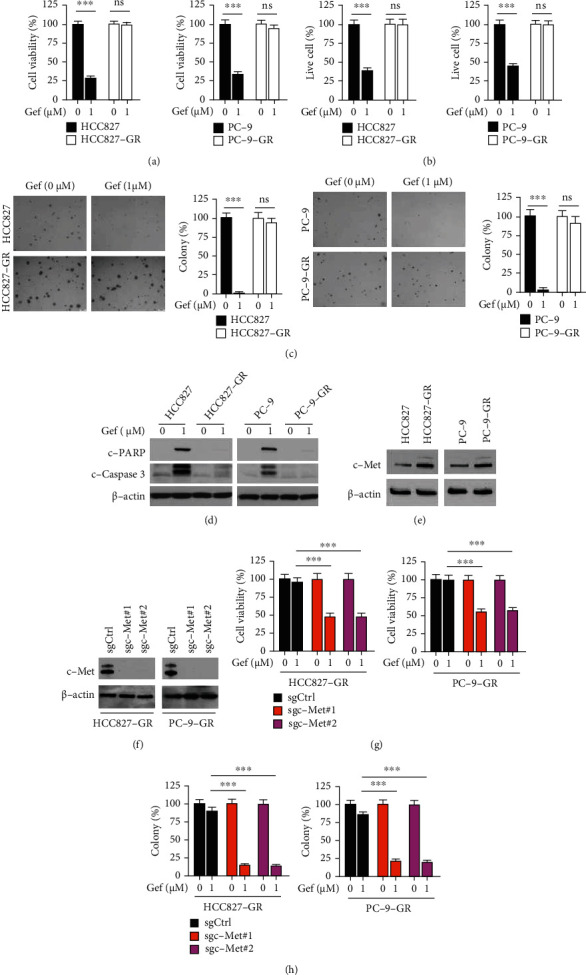
c-Met is overexpressed in gefitinib resistant cells. (a) HCC827/HCC827-GR (left) and PC-9/PC-9-GR (right) cells were treated with gefitinib for 72 h, cell viability was examined by MTS assay. ^∗∗∗^*p* < 0.001. ns: not statistically significant. Gef: gefitinib. (b) Trypan blue exclusion assay analyzes live cell population of HCC827/HCC827-GR (left) and PC-9/PC-9-GR (right) cells treated with gefitinib for 72 h. ^∗∗∗^*p* < 0.001. (c) HCC827/HCC827-GR (left) and PC-9/PC-9-GR (right) cells were treated with gefitinib for 72 h, colony formation was tested by soft agar assay. ^∗∗∗^*p* < 0.001. (d) HCC827/HCC827-GR (left) and PC-9/PC-9-GR (right) cells were treated with gefitinib for 72 h, whole cell extract (WCE) was subjected to immunoblotting (IB) analysis. (e) IB analysis of c-Met protein level in HCC827/HCC827-GR (left) and PC-9/PC-9-GR (right) cells. (f–h) c-Met-null HCC827-GR (left) and PC-9-GR (right) cells were treated with gefitinib for 72 h, c-Met expression was examined by IB analysis (f), cell viability (g), and colony formation (h) was tested via MTS assay and soft agar assay, respectively. ^∗∗∗^*p* < 0.001.

To further discover novel antitumor agents that can overcome gefitinib resistance, two gefitinib-resistant cell lines were generated, namely HCC827-GR and PC-9-GR. The MTS data indicated that gefitinib significantly decreased the viability of HCC827 and PC-9 cells but not that of HCC827-GR and PC-9-GR cells (Figure 1(a)). The trypan blue exclusion assay suggested that the viable cell population of HCC827-GR and PC-9-GR cells was not significantly decreased (Figure 1(b)). Furthermore, the colony formation ability of gefitinib-resistant NSCLC cells was examined. The results indicated that the colony formation numbers of HCC827 and PC-9 cells were markedly suppressed with gefitinib treatment. In contrast to these observations, gefitinib did not reduce the colony formation numbers of HCC827-GR and PC-9-GR cells (Figure 1(c)). The protein expression levels of cleaved poly (ADP-ribose) polymerase (PARP) and cleaved-caspase 3 were notably decreased in gefitinib-treated HCC827-GR and PC-9-GR cells, as determined via IB analysis (Figure 1(d)). To investigate whether c-Met is associated with gefitinib resistance, its protein expression levels were examined in HCC827/HCC827-GR and PC-9/PC-9-GR cells. IB indicated that the expression levels of the c-Met protein in HCC827-GR and PC-9-GR cells were upregulated compared with those of HCC827 and PC-9 cells (Figure 1(e)). Moreover, the knockdown of c-Met (Figure 1(f)) promoted the antitumor effect of gefitinib in resistant cells, as determined by the significant decrease noted in the viability and colony formation activity of HCC827-GR and PC-9-GR cells (Figures 1(g) and 1(h)). These results suggested that c-Met was required for gefitinib resistance in HCC827-GR and PC-9-GR cells.

### 3.2. Lico A Inhibits the Viability of Gefitinib-Resistant NSCLC Cells

**Figure 2:**
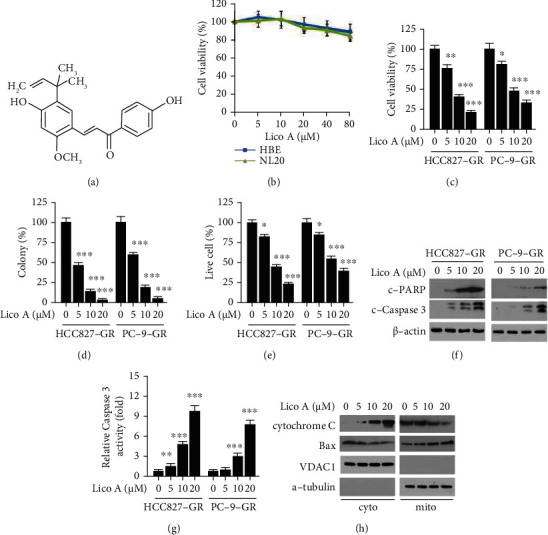
Licochalcone A (Lico A) overcomes gefitinib resistance. (a) The chemical structure of Lico A. (b) The cytotoxicity of Lico A on immortalized HBE and NL20 cells. (c and d) HCC827-GR (left) and PC-9-GR (right) cells were treated with Lico A for 48 h, cell viability (c) and colony formation (d) was examined by MTS and soft agar assay, respectively. ^∗^*p* < 0.05, ^∗∗^*p* < 0.01, and ^∗∗∗^*p* < 0.001. (e) HCC827-GR (left) and PC-9-GR (right) cells were treated with Lico A for 48 h, trypan blue exclusion assay was performed to analyze the live cell population. ^∗^*p* < 0.05 and ^∗∗∗^*p* < 0.001. (f and g) HCC827-GR (left) and PC-9-GR (right) cells were treated with Lico A for 48 h, WCE was subjected to IB analysis (f), caspase 3 activity was measured using the Caspase-3 Assay Kit (g). ^∗∗^*p* < 0.01 and ^∗∗∗^*p* < 0.001. (h) HCC827-GR cells were treated with Lico A for 48 h, subcellular fractions were isolated and subjected to IB analysis.

Earlier studies demonstrated that Lico A exerted a wide range of pharmacological effects ranging from anti-inflammatory to antitumor modes of action (Figure 2(a)). However, the inhibitory effects of Lico A on gefitinib-resistant NSCLC and the underlying mechanism remain elusive. The present study investigated whether Lico A exerted cytotoxic effects on immortalized non-tumor lung epithelial cells. Lico A exhibited no apparent inhibitory effects on HBE and NL20 cells (Figure 2(b)). In contrast to these findings, the MTS results indicated that Lico A suppressed the viability of gefitinib-resistant HCC827-GR and PC-9-GR cells dose-dependently. Gefitinib attenuated the viability of HCC827-GR and PC-9-GR cells at ~75% following treatment with 5 *μ*M Lico A for 48 h. Exposure to higher concentrations (10 or 20 *μ*M) of Lico A exhibited a more potent inhibitory effect (Figure 2(c)). The soft agar data indicated that Lico A markedly reduced the colony formation number of HCC827-GR and PC-9-GR cells in a dose-dependent manner (Figure 2(d)). Furthermore, the trypan blue exclusion assay indicated that Lico A reduced the viable cell population following exposure to different concentrations of this compound for 48 h (Figure 2(e)). Lico A increased the protein expression levels of cleaved PARP and cleaved caspase 3 (Figure 2(f)). The relative enzyme activity of caspase 3 was augmented following an increase in Lico A concentration (Figure 2(g)). In addition, IB analysis indicated that Lico A promoted the release of cytochrome c from the mitochondria to the cytoplasm. In addition, this compound dose-dependently augmented the expression of Bax in the mitochondrial fraction (Figure 2(h)), suggesting that it could activate the intrinsic apoptotic pathway. These results suggested that Lico A substantially suppressed the proliferation of gefitinib-resistant NSCLC cells and induced their apoptosis.

### 3.3. Lico A Suppresses c-Met Signaling

**Figure 3:**
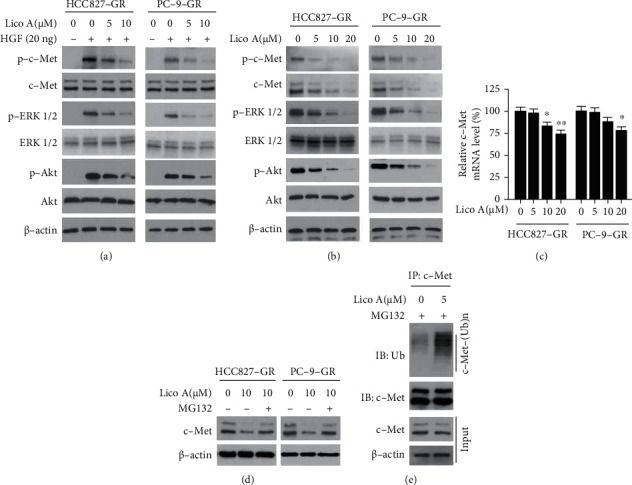
Lico A inhibits c-Met signaling. (a) HCC827-GR (left) and PC-9-GR (right) cells were starved overnight with 0.1% FBS, and pretreated with Lico A for 4 h. Cells were stimulated with 20 ng HGF for 5 min, WCE was prepared and subjected to IB analysis. (b) HCC827-GR (left) and PC-9-GR (right) cells were treated with Lico A for 48 h, WCE was subjected to IB analysis. (c) qRT-PCR analyzes the mRNA levels of c-Met in Lico A-treated HCC827-GR (left) and PC-9-GR (right) cells. ^∗^*p* < 0.05 and ^∗∗^*p* < 0.01, (d) HCC827-GR (left) and PC-9-GR (right) cells were treated with Lico A for 48 h, followed by incubation with MG132 for 6 h, WCE was subjected to IB analysis. (e) HCC827-GR cells were treated with Lico A for 48 h, followed by incubation with MG132 for 6 h, WCE was subjected to immunoprecipitation (IP) and IB analysis.

Subsequently, the ability of Lico A to affect c-Met signaling was examined. IB analysis indicated that short-term exposure (4 h) to Lico A inhibited hepatocyte growth factor- (HGF-) induced c-Met phosphorylation. Moreover, the activation of the c-Met downstream kinases, ERK1/2 and AKT, was substantially reduced. However, the total protein levels of c-Met were unaffected (Figure 3(a)). The data further demonstrated that long-term exposure to Lico A decreased c-Met phosphorylation and its total protein levels in a dose-dependent manner (Figure 3(b)). These results indicated that Lico A attenuated the activation of c-Met signaling in gefitinib-resistant NSCLC cells. Reverse transcription-quantitative PCR data revealed that Lico A decreased the mRNA levels of c-Met when 10 and 20 *μ*M of this compound were added to HCC827-GR and PC-9-GR cells, respectively (Figure 3(c)). This suggested that Lico A slightly suppressed c-Met transcription to some extent (Figure 3(c)). Moreover, exposure to the proteasome inhibitor, MG132 restored c-Met expression in Lico A-treated HCC827-GR and PC-9-GR cells (Figure 3(d)). To further validate that Lico A promoted c-Met degradation, the ability of this compound to induce c-Met ubiquitination was examined in gefitinib-resistant NSCLC cells. Exposure of HCC827-GR cells to Lico A enhanced the endogenous ubiquitination of c-Met (Figure 3(e)). These results suggested that Lico A suppressed c-Met signaling and promoted c-Met ubiquitination.

### 3.4. c-Cbl is Required for Lico A-Induced c-Met Ubiquitination

**Figure 4:**
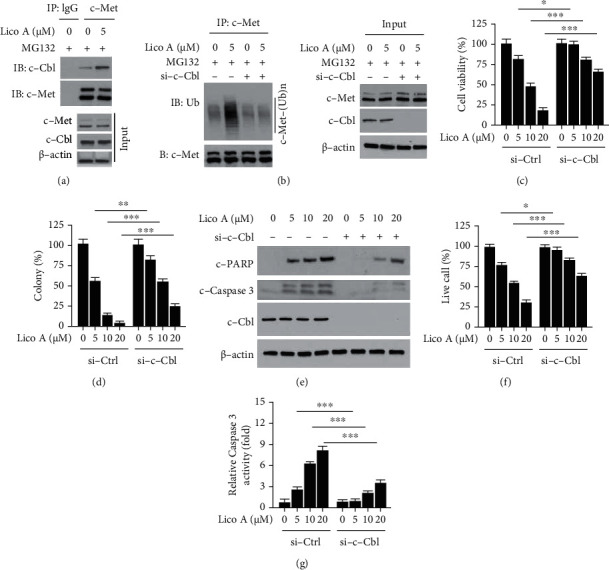
c-Cbl is required for Lico A-induced c-Met degradation. (a) HCC827-GR cells were treated with Lico A for 48 h, followed by incubation with MG132 for another 6 h. Cell lysates were subjected to co-immunoprecipitation (co-IP) analysis. (b) HCC827-GR cells were transfected with si-c-Cbl for 24 h, followed by treated with Lico A for 48 h. Cells were incubated with MG132 for 6 h, WCE was collected and subjected to IP and IB analysis. (c and d) HCC827-GR cells were transfected with si-c-Cbl for 24 h, followed by treated with Lico A for 48 h. Cell viability (c) and colony formation (d) was analyzed by MTS and soft agar assay, respectively. ^∗^*p* < 0.05, ^∗∗^*p* < 0.01, and ^∗∗∗^*p* < 0.001. (e–g) HCC827-GR cells were transfected with si-c-Cbl for 24 h, followed by treated with Lico A for 48 h. WCE was subjected to IB analysis (e), trypan blue exclusion assay (f) and Caspase-3 Assay Kit (g) was used for live cell population examination and caspase-3 activity measurement, respectively. ^∗^*p* < 0.05 and ^∗∗∗^*p* < 0.001.

Previous studies have shown that the E3 ligase c-Cbl promotes the degradation of c-Met ubiquitination. Therefore, it was hypothesized that c-Cbl was involved in Lico A-induced c-Met ubiquitination. The interaction between c-Cbl and c-Met was initially determined. The results indicated that Lico A strengthened the interaction between c-Cbl and c-Met in HCC827-GR cells (Figure 4(a)). Moreover, IB analysis indicated that c-Cbl knockdown prominently compromised Lico A-enhanced c-Met ubiquitination (Figure 4(b)). Moreover, knockdown of c-Cbl rescued Lico A-suppressed viability and colony formation activity in HCC827-GR cells (Figures 4(c) and 4(d)). In addition, we investigated what effect c-Cbl knockdown exerted on PARP cleavage and release of cleaved-caspase 3 in HCC827-GR cells, which are considered markers of apoptosis (Figure 4(e)). Lico A enhanced the protein levels of cleaved-PARP and cleaved-caspase 3 dose-dependently in c-Cbl sufficient cells (Figure 4(e)). Conversely, c-Cbl knockdown reduced the protein levels of cleaved-PARP and cleaved-caspase 3. Moreover, knockdown of c-Cbl notably increased the viable cell population in Lico A-treated HCC827-GR cells, while the activity of caspase 3 was suppressed by c-Cbl knockdown (Figures 4(f) and 4(g)). Overall, these results suggested that c-Cbl was required for Lico A-induced c-Met ubiquitination in gefitinib-resistant HCC827-GR cells.

### 3.5. Lico A Overcomes Gefitinib Resistance In Vivo

**Figure 5:**
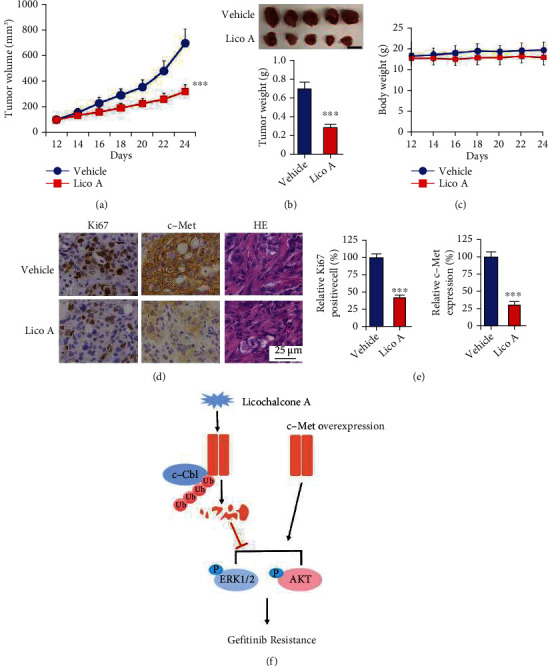
Lico A overcomes gefitinib resistance in vivo. (a) The tumor volumes of HCC827-GR-derived xenograft tumors with vehicle or Lico A treatment. ^∗∗∗^*p* < 0.001. (b) Tumor weight of vehicle- or Lico A-treated xenograft tumors. ^∗∗∗^*p* < 0.001. Scale bar, 1 cm. (c) The body weight of tumor-bearing mouse with vehicle or Lico A treatment. (d) Immunohistochemical staining analysis of ki67 and c-Met in vehicle- or Lico A-treated xenograft tumors. (e) Qualification analysis of ki67 and c-Met staining from (d). ^∗∗∗^*p* < 0.001. Scale bar, 25 *μ*M. (f) A schematic illustration representing Licochalcone A inhibits c-Met signaling by promoting c-Cbl-mediated c-Met ubiquitination.

To verify the in vivo inhibitory effect of Lico A, a xenograft mouse model was established. HCC827-GR-derived xenograft tumors were treated with Lico A and vehicle control. The data indicated that Lico A significantly delayed the tumor growth of HCC827-GR xenografts compared with that of the vehicle control (Figure 5(a)). The average tumor weight of the Lico A-treated tumors was substantially lower than that of vehicle-treated groups (Figure 5(b)). In addition, the bodyweight of Lico A-treated mice did not exhibit a significant decrease compared with that of the vehicle-treated groups (Figure 5(c)). The IHC staining analysis was performed to examine the in vivo inhibitory effect of Lico A on c-Met expression. The population of Ki67-positive cells was reduced with Lico A treatment, indicating that this compound could inhibit cell proliferation in vivo. In addition, the positive staining of c-Met was suppressed in Lico A-treated HCC827-GR xenograft tumors (Figures 5(d) and 5(e)). These results suggested that Lico A inhibited the in vivo tumor growth of gefitinib-resistant xenografts.

## 4. Discussion

NSCLC is the leading cause of cancer-associated deaths worldwide and accounts for ~85% of lung cancer cases. Lung squamous carcinomas and lung adenocarcinomas are the main subtypes of NSCLC [1, 2]. EGFR mutations have been detected in 40–50% of lung adenocarcinomas [6, 30, 31]. Patients with NSCLC harboring EGFR activating mutations benefit from the clinical application of EGFR TKIs, such as gefitinib [6, 10, 32]. Unfortunately, acquired resistance to EGFR-TKIs is inevitable and develops after a median of 9.2–14.7 months of TKI therapy [7, 13, 33]. Previous studies have demonstrated that the EGFR T790M mutation is the most common mechanism of acquired resistance to gefitinib [34–36]. It is interesting to note that the amplification of the MET gene contributes to the resistance of NSCLC cells to gefitinib. This gene encodes the c-Met tyrosine kinase receptor, and its amplification is associated with deregulated c-Met expression levels [37–40]. The occurrence of the T790M mutation and the MET amplification account for 70% of acquired resistance to gefitinib in NSCLC [34]. At present, no specific targeted therapy strategies have been reported for EGFR-mutant NSCLC cases who develop resistance to EGFR TKIs based on Met proto-oncogene amplification [6, 41]. The evaluation of the c-Met inhibitor tepotinib in combination with the EGFR TKI gefitinib is ongoing to assess their therapeutic efficacy [37, 42]. Therefore, identifying novel small molecule inhibitors that can overcome gefitinib resistance is still an urgent demand for NSCLC treatment.

Natural products are widely studied as potential therapeutic antitumor agents due to their limited toxicity [43]. Lico A is a novel flavonoid extracted from licorice root [23]. Lico A efficiently exerts roles of anti-inflammation and antitumor without significant side effects and drug toxicity. Relevant studies indicated that the administration of Lico A alone significantly reduced the size of the solid tumors in Balb/c mice without any detectable induction of nephrotoxicity and hepatotoxicity [44]. Lico A had no adverse effect on HFF cell viability at concentrations below 9 *μ*g/ml [45]. Concentrations of 147.75 *μ*M or higher Lico A produced cytotoxicity in Chinese hamster ovary (CHO) fibroblasts. Lower concentrations (1.85 to 7.39 *μ*M) exhibited protective activity against chromosomal damage induced by doxorubicin (DXR) or methyl methanesulfonate (MMS) in CHO cells [46]. While applying selective Met inhibitors tepotinib or capmatinib, some clinical trials revealed peripheral edema and nausea were the main toxic effects [37, 47]. Thus, Lico A or the derivative is a potential candidate that deserves further cancer treatment study. The present study demonstrated that Lico A dose-dependently induced intrinsic apoptosis and exhibited antiproliferative efficacy against gefitinib-resistant NSCLC cells.

Licochalcone B and Licochalcone D, which are structurally similar derivatives to Lico A, induce apoptosis by dual inhibition of EGFR and MET expression. Licochalcone B and Licochalcone D inhibited both EGFR and MET kinase activity by competing with their ATP-binding pockets [48, 49]. Moreover, Lico A was suggested as an Hsp90 inhibitor to inhibit H1975 cells. Our results revealed that Lico A promoted c-Cbl-mediated c-Met ubiquitination in gefitinib-resistant HCC827-GR and PC-9-GR cells (Figure 5(f)). Consequently, Lico A dose-dependently reduced c-Met protein level, as well as the phosphorylation of c-Met, ERK1/2, and AKT. The depletion of c-Cbl compromised the inhibitory effect of Lico A against gefitinib-resistant NSCLC cells. Our study firstly elucidated that Lico A promoted the ubiquitination of c-Met and revealed a novel antitumor mechanism of the natural product Lico A in NSCLC, indicating that activation of ubiquitination-mediated protein degradation signaling might be a promising antitumor strategy to overcome gefitinib resistance. Our study further manifests that Lico A is a potential candidate for targeted protein degradation and that the E3 ligase c-Cbl and c-Met can be targeted for PROTAC drug discovery to more efficiently cure cancer.

In addition to the secondary mutation in EGFR, multiple EGFR-independent resistance mechanisms have been identified, including the bypass receptor tyrosine kinases (RTKs) activation (e.g. Met, HER2 amplification, and AXL pathway activation), hyperactivation of downstream signaling (e.g. PI3K and BRAF mutations), and histological transformations (e.g. small cell transformation and epithelial-mesenchymal transition) [50]. Moreover, tumor heterogeneity occurs with different oncogenic driver mutations or resistance mechanisms. The different resistant mutations may occur at a small clone of tumor cells, and clonal evolution may develop during the EGFR-TKIs treatment process [7, 51]. EGFR T790M mutation can co-occur with ERBB2 and/or MET amplification, and AXL expression can increase epithelial-mesenchymal transition. These co-occurring mutations possibly influence the treatment outcomes [6]. However, our study does not have the sequence data on the mechanism underlying the gefitinib acquired resistance in these two cell lines. It remains unknown whether other EGFR-dependent or -independent resistant mechanisms synergistically induce gefitinib resistance with c-Met activation. Also, the inhibitory effect of Lico A on the crosstalk between different resistance mechanisms needs further addressed. In addition, whether other deubiquitinases (DUBs) stabilizing c-Met and other related RTKs proteins participate in resistance mechanisms and what effects Lico A exerts on DUBs to antagonize acquired resistance, and TKIs also need further exploration to fill the gap in the future.

In summary, the present study demonstrated that the natural product Lico A inhibited c-Met signaling and disrupted c-Met ubiquitination in a c-Cbl-dependent manner. Targeting c-Met degradation is a promising strategy to abrogate gefitinib resistance in NSCLC cells. The current study provided new insights on the role of Lico A in NSCLC treatment and suggested that this compound may be a promising therapeutic agent for gefitinib-resistant NSCLC.

## Data Availability

The data used to support the findings of this study are included within the article and figure legend.

## Abbreviations

NSCLC: Non-small cell lung cancer
c-Met: Cellular-mesenchymal to epithelial transition factor
EGFR: Epidermal growth factor receptor
ROS: Reactive oxygen species
HGF: Hepatocyte growth factor
ERK: Extracellular regulated protein kinases
AKT: Protein kinase B
KRAS: Kirsten rat sarcoma
ALK: Anaplastic lymphoma kinase
PIK3CA: Phosphatidylinositol-4,5-bisphosphate 3-Kinase catalytic subunit alpha
EMT: Epithelial-mesenchymal transition
TKIs: Tyrosine kinase inhibitors
DUBs: Deubiquitinases
WCE: Whole-cell extract
IB: Immunoblotting
IHC: Immunohistochemical
SD: Standard deviation.
